# AlGaN/GaN on SiC Devices without a GaN Buffer Layer: Electrical and Noise Characteristics

**DOI:** 10.3390/mi11121131

**Published:** 2020-12-20

**Authors:** Justinas Jorudas, Artūr Šimukovič, Maksym Dub, Maciej Sakowicz, Paweł Prystawko, Simonas Indrišiūnas, Vitalij Kovalevskij, Sergey Rumyantsev, Wojciech Knap, Irmantas Kašalynas

**Affiliations:** 1 Center for Physical Sciences and Technology (FTMC), Saulėtekio 3, 10257 Vilnius, Lithuania; arturas.simukovic@ftmc.lt (A.Š.); simonas.indrisiunas@ftmc.lt (S.I.); vitalij@ftmc.lt (V.K.); 2 Institute of High Pressure Physics PAS, ul. Sokołowska 29/37, 01-142 Warsaw, Poland; mdub@unipress.waw.pl (M.D.); sakowicz400@gmail.com (M.S.); pprysta@unipress.waw.pl (P.P.); roumis4@gmail.com (S.R.); knap.wojciech@gmail.com (W.K.); 3 CENTERA Laboratories, Institute of High Pressure Physics PAS, ul. Sokołowska 29/37, 01-142 Warsaw, Poland

**Keywords:** AlGaN/GaN, SiC, high electron mobility transistor, Schottky barrier diode, breakdown field, noise, charge traps, radio frequency

## Abstract

We report on the high-voltage, noise, and radio frequency (RF) performances of aluminium gallium nitride/gallium nitride (AlGaN/GaN) on silicon carbide (SiC) devices without any GaN buffer. Such a GaN–SiC hybrid material was developed in order to improve thermal management and to reduce trapping effects. Fabricated Schottky barrier diodes (SBDs) demonstrated an ideality factor *n* at approximately 1.7 and breakdown voltages (fields) up to 780 V (approximately 0.8 MV/cm). Hall measurements revealed a thermally stable electron density at *N*_2*DEG*_ = 1 × 10^13^ cm^−2^ of two-dimensional electron gas in the range of 77–300 K, with mobilities *μ* = 1.7 × 10^3^ cm^2^/V∙s and *μ* = 1.0 × 10^4^ cm^2^/V∙s at 300 K and 77 K, respectively. The maximum drain current and the transconductance were demonstrated to be as high as 0.5 A/mm and 150 mS/mm, respectively, for the transistors with gate length *L_G_* = 5 μm. Low-frequency noise measurements demonstrated an effective trap density below 10^19^ cm^−3^ eV^−1^. RF analysis revealed *f_T_* and *f_max_* values up to 1.3 GHz and 6.7 GHz, respectively, demonstrating figures of merit *f_T_* × *L_G_* up to 6.7 GHz × µm. These data further confirm the high potential of a GaN–SiC hybrid material for the development of thin high electron mobility transistors (HEMTs) and SBDs with improved thermal stability for high-frequency and high-power applications.

## 1. Introduction

Aluminium gallium nitride/gallium nitride (AlGaN/GaN) high electron mobility transistors (HEMTs) are widely used in high-power and high-frequency applications due to their superior characteristics based on the unique physical properties of III-nitride materials. The AlGaN/GaN heterostructures can be grown on sapphire, silicon, silicon carbide, and native GaN substrates [1,2,3,4,5,6,7]. While sapphire and silicon substrates are the most cost-effective, the best characteristics are achieved on transistors fabricated on silicon carbide (SiC) and GaN substrates. Considerable improvements in electrical performance including the low-frequency noise were demonstrated on the AlGaN/GaN/sapphire platform [8,9]. The advantage of the SiC over GaN substrates is higher SiC thermal conductivity and therefore potentially better thermal management of the transistors fabricated using AlGaN/GaN/SiC structures. The common approach to compensate for lattice mismatch and to reduce the dislocation density in these structures is to grow the aluminium gallium nitride (AlN) nucleation layer (NL) with reduced crystalline quality followed by a several-micrometres-thick GaN buffer doped with deep acceptors such as Fe or C which compensate for residual doping of an *n*-type GaN [10,11,12]. However, this approach deteriorates the overall thermal resistance of the structure and diminishes the advantage of a SiC substrate operating as a heatsink [13,14]. Also, the acceptor-type impurities in a thick GaN buffer introduce deep charge trapping centres, resulting in the increase of low-frequency noise, and facilitate the current collapse effects in HEMTs [9,15,16].

A new heteroepitaxy approach based on thin GaN–AlN–SiC heterostructures without a GaN buffer has been developed recently [17,18]. Although this approach has already been demonstrated to be promising, there are only a few studies on realistic devices such as transistors [17,19]. Thin GaN–AlN–SiC structures should provide better thermal management of the devices and could potentially reduce short channel effects. One expects also that this technology will reduce the effects of traps from a GaN:C buffer. However, the GaN:C buffer helps in reducing the number of threading dislocations. Therefore, GaN–AlN–SiC structures with the thin buffer may exhibit a higher concentration of threading dislocations, which may deteriorate the characteristics of devices. Indeed, it is well known that the dislocations may act as traps increasing low-frequency noise and current collapse effects and/or lowering maximum voltage breakdown of the devices.

In this work, the GaN–AlN–SiC hybrid material was used to develop thin Schottky barrier diodes (SBDs) and thin HEMTs (T-HEMTs) to study realistic devices under high DC voltages and in radio frequency (RF) regimes. We show that all the devices fabricated on this material have good thermal stability and demonstrate good DC as well as radio frequency (RF) characteristics. By systematic low-frequency noise measurements, we estimated the trap density, showing that avoiding a GaN:C buffer in the GaN–AlN-SiC material does not lead to an increase in active (dislocation related) trap density. We also show that deep trap-related current collapse phenomena are avoided and that all the fabricated devices demonstrate good DC, high voltage, as well as radio frequency (RF) characteristics. This way, we confirm the high potential of a GaN–SiC hybrid material in the development of improved thermal stability HEMTs and SBDs for high-frequency and high-power applications.

## 2. Materials and Methods (Experimental Details)

**Figure 1:**
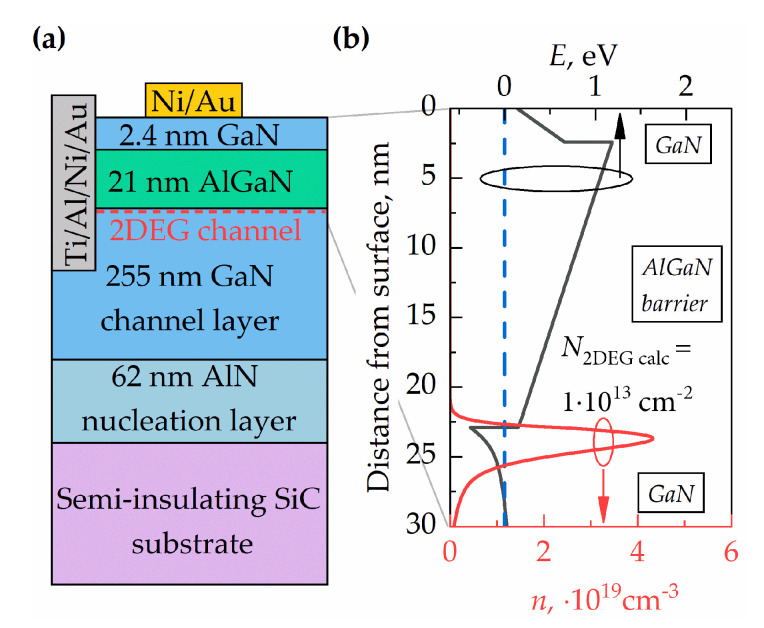
(**a**) Schematic of the thin high electron mobility transistor (T-HEMT) structure cross section with ohmic and Schottky contacts and (**b**) the calculated band diagram and electron density distribution in the upper layers of the heterostructure.

The heterostructures with the sequence of layers shown in Figure 1a were obtained commercially from the “SweGaN” company. They were grown on a 4” diameter, 500-μm-thick semi-insulating SiC substrate. The layers consisted of a 2.4-nm GaN cap, a 20.5-nm Al_0.25_Ga_0.75_N barrier, and a 255-nm GaN channel grown directly on a 62-nm high-quality AlN NL on SiC. The sheet resistance (*R_Sh_*) of the as-grown T-HEMT structure determined from contactless eddy current measurements was 380 ± 10 Ω/□. The band diagram and electron distribution were calculated by a 1D Poisson simulator using the nominal thickness of all layers [20,21]. The results are shown in Figure 1b. The density of the two dimensional electron gas (2DEG) was calculated by integrating an electron distribution in the quantum well. Its value was found to be about 1 × 10^13^ cm^−2^.

The devices were fabricated using standard ultraviolet (UV) photolithography [8,22]. Mesas of 140 nm depth were formed by inductively coupled plasma reactive ion etching (ICP-RIE) (Oxford Instruments, Bristol, UK) using Cl plasma and chemical treatment in tetramethylammonium hydroxide (TMAH) solution (Microchemicals, Ulm, Germany). For ohmic contacts, Ti/Al/Ni/Au metal stacks of thicknesses 30/90/20/150 nm were deposited and annealed in nitrogen ambient for 30 s at 850 °C. The resistance (*R_c_*), and the specific resistivity (*ρ_c_*) of ohmic contacts were determined by transmission line method (TLM), demonstrating average values of about 1 Ω × mm and 2 × 10^−5^ Ω × cm^2^, respectively. Schottky contacts were formed from Ni/Au (25/150 nm).

**Figure 2:**
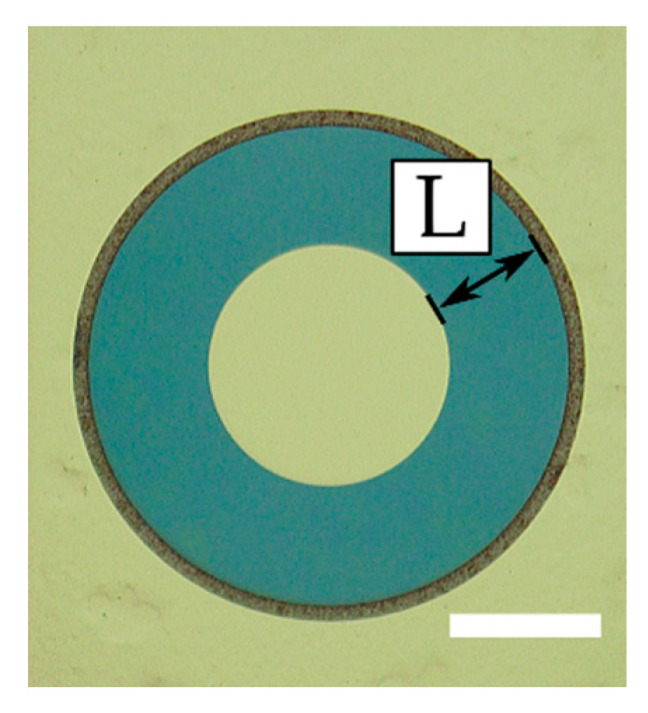
Microscope image of the fabricated Schottky barrier diode (SBD): *L* is the separation between the Ohmic and Schottky contacts. The scale bar is 50 μm.

**Figure 3:**
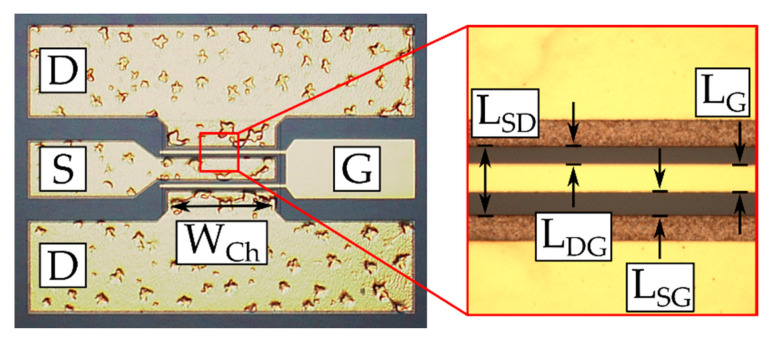
Microscope image of the radio frequency (RF) T-HEMT (left hand side) and details of the design parameters (right hand side) illustrating the Gate (G), Source (S), and Drain (D) electrodes in a 150-μm pitch implementation.

**Figure 4:**
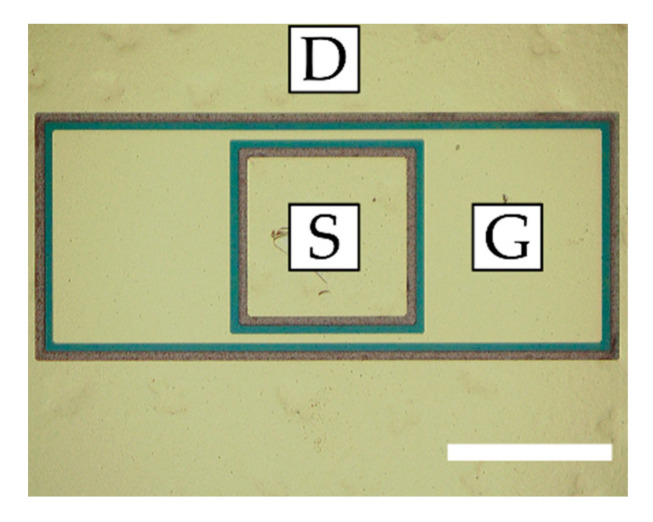
Microscope image of the fabricated DC T-HEMT: the scale bar is 100 μm.

The Schottky diodes (Figure 2) and HEMTs of two different designs (see Figure 3 and Figure 4) were fabricated. Circular SBDs were used by depositing an inner Schottky contact with radius *r* = 40 µm and an outer ohmic contact of a variable radius in such a way that the distance between the electric contact, *L*, ranged from 5 µm to 40 µm (see Figure 2). The designs of the Schottky diodes and transistors shown in Figure 2 and Figure 4, respectively, do not require mesa isolation. For testing at RF, the transistor design shown in Figure 3 was used (RF T-HEMT).

These RF T-HEMTs consisted of 200 μm (RF T-HEMT-1) or 300 μm (RF T-HEMT-2)-wide two-finger transistors, each of drain-source distance *L_SD_* = 14 μm, gate length *L_G_* = 5 μm, and gate-source distance *L_SG_* = 5 μm. For comparison, the T-HEMTs with rectangular-type electrodes (see Figure 4), labelled here as DC T-HEMT, were also investigated (see also reference [9]). Similar to RF T-HEMTs, all DC T-HEMTs had the same gate length and gate-source distance of 5 μm, but the channel width was of 200 μm and the drain-source distances were 17.5 μm, 15 μm, and 12.5 μm for three sample transistors labelled DC T-HEMT-1, DC T-HEMT-2, and DC T-HEMT-3, respectively.

All transistors were measured on the wafer in DC and RF regimes by using Süss Microtech probe station PM8 (SUSS MicroTec SE, Garching, Germany). For the RF measurements, the G-S-G (ground–signal–ground) 150-μm pitch high frequency probes, Agilent E8364B PNA Network Analyzer (Agilent, Santa Clara, CA, USA), and E5270B Precision IV Analyzer with IC-CAP software were used (Keysight Technologies, Santa Rosa, CA, USA). The two-step open-short de-embedding method was implemented, and small signal S-parameters were obtained. The unity current gain cut-off frequency (*f_T_*) and the unity maximum unilateral power gain frequency (*f_max_*) were found from de-embedded S-parameter frequency characteristics.

The SBDs were investigated using EPS150 probe station (Cascade Microtech, Beaverton, OR, USA), high voltage source-meter Keithley 2410 (Tektronix, Beaverton, OR, USA), and impedance analyser Agilent 4294A (Agilent, Santa Clara, CA, USA).

The low-frequency noise in transistors was measured in the linear regime with the source grounded. The voltage fluctuations from the drain load resistor, *R_L_*, were amplified by a low-noise amplifier and analysed using “PHOTON” spectrum analyser (Bruel & Kjaer, Nærum, Denmark). The spectral noise density of drain current fluctuations was calculated in the usual way with $S_{I}=S_{V}{\left(\left(R_{L}+R_{DS}\right)/R_{L}R_{DS}\right)}^{2}$, where *S_V_* is the drain voltage fluctuations and *R_DS_* is the total drain to source resistance.

## 3. Experimental Results and Discussions

The 2DEG density (*N*_2*DEG*_), mobility (*μ*_2*DEG*_), and sheet resistance (*R_Sh_*) were determined in the Hall experiments using Van der Pauw (VdP) geometry. The results are summarized in Table 1. Good agreement between the calculated carrier density, an integral of electron distribution in the quantum well (see Figure 1b), measured sheet resistance using contactless eddy current method, and the results of the Hall experiment were found within a deviation interval of 7%.

These values are typical for the state-of-the-art AlGaN/GaN heterostructures [23,24,25,26,27]. Therefore, we can conclude that elimination of the buffer layer did not worsen the parameters of the 2DEG.

### 3.1. Performance of SBDs

**Figure 5:**
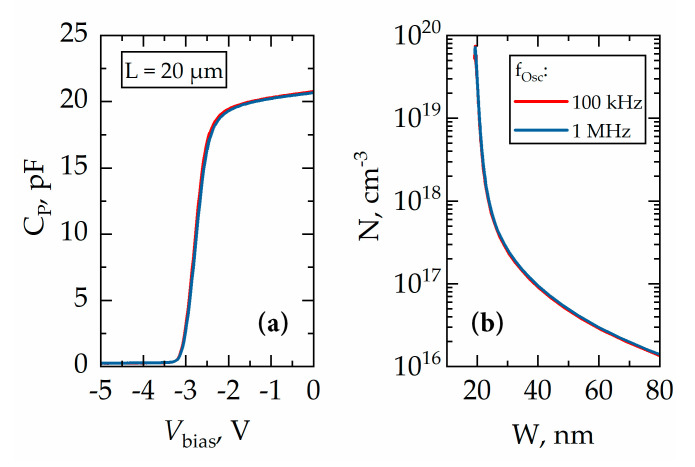
(**a**) Capacitance-voltage (*C*-*V*) characteristics of SBD with *L* = 20 μm at modulation frequencies of 100 kHz (red line) and 1 MHz (blue line), and (**b**) carrier distribution *N(W)* calculated from *C*-*V* data using Equations (2) and (3).

Typical capacitance–voltage (*C*-*V*) characteristics of SBD measured at frequencies 100 kHz and 1 MHz are shown in Figure 5a. One can see that frequency dispersion is negligible, indicating that deep levels do not affect the *C*-*V* characteristics. The pinch-off voltage (*V_po_*) needed to fully deplete a 2DEG channel was found to be about −3.1 V. The density of 2DEG under Schottky contact was calculated using the integral capacitance technique [28]:

(1) $$N_{G-2DEG}=\frac{1}{eA}\int_{V_{po}}^{0}C_{P}(V)dV,$$

where *e* is the elementary charge, *A* is the area of Schottky contact, and *C_P_*(*V*) is the capacitance. The carrier density *N* dependence on the distance from the surface *W* was found from *C*-*V* data using the following formulas [28]:

(2) $$W=\frac{\varepsilon \varepsilon_{0}A}{C_{P}},$$

(3) $$N=\frac{C_{P}{}^{3}}{e\varepsilon \varepsilon A^{2}}{\left(\frac{dC_{P}(V)}{dV}\right)}^{-1},$$

where *ε* = 8.9 is the relative permittivity of GaN and *ε_0_* is the vacuum permittivity. The obtained *N* dependence on the parameter *W* is shown in Figure 5b. The density of 2DEG was found to be *N*_*G*-2*DEG*_ = 0.69 × 10^13^ cm^−2^ at 300 K. This density is smaller than that found from the Hall measurements due to depletion by the Schottky barrier built-in voltage [29,30].

**Figure 6:**
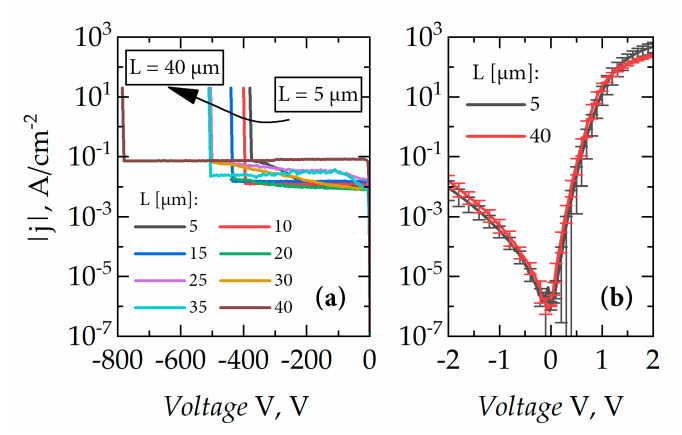
(**a**) Reverse current-voltage characteristics of SBDs and (**b**) current-voltage characteristics of SBDs with *L* = 5 and 40 μm at low voltages.

Figure 6 shows examples of the forward and reverse current–voltage characteristics of SBDs. The forward current–voltage characteristics demonstrated an ideality factor of *n* ≅ 1.7. The barrier height found based on the thermionic emission (TE) model was *φ* = 0.75 eV. These values are typical for Ni/AlGaN Schottky barriers [31]. Under reverse bias, leakage currents were saturated at approximately −5 V and remained constant until the breakdown (see Figure 6b). Moreover, SBDs demonstrated a sufficiently high *j_ON_*/*j_OFF_* ratio; for example, for SBD with *L* = 40 µm, the highest achieved value was found to be more than three orders of magnitude, *j_ON_*/*j_OFF_* ≥ 3200, taking into account also the reverse-current densities prior to a breakdown which occurred at a voltage of −780 V. Furthermore, a 2.5 times improvement in the maximum current density was obtained in comparison with previously reported SBDs fabricated on standard AlGaN/GaN HEMT structures with a thick GaN:C buffer [8]. Note the dependence of forward current on the distance between ohmic and Schottky contacts indicating good performance of the fabricated ohmic contacts with negligible losses.

**Figure 7:**
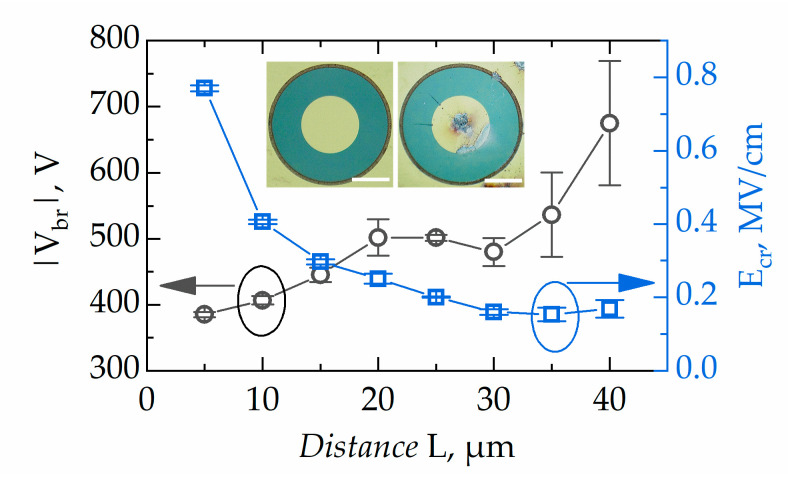
Breakdown voltage and critical electric field dependences on the distance between ohmic and Schottky contacts: *error bars in the critical field data are depicted by the size of the symbols*. *Inset:* images of *L* = 40 μm SBD before and after breakdown (scale bar is 50 μm).

GaN–AlN–SiC buffer-free structures with a thin AlN layer may potentially exhibit a higher concentration of the threading dislocations, which may deteriorate the breakdown characteristics. On the other hand, as discussed in References [17,18], high-quality AlN NL in a T-HEMT structure can serve as a back barrier which enhances the critical breakdown field. Figure 7 shows the breakdown voltage and critical electric field dependences on the distance between ohmic and Schottky contacts.

As seen in Figure 7, the breakdown voltage depends on the distance, *L*, between contacts and ranges from 800 V to 400 V for *L* = 40 μm and *L* = 5 μm, respectively. The average breakdown field for *L* = 5 μm devices was found to be 0.8 MV/cm. It is worth noting that the maximum critical field asymptotically decreased down to 0.2 MV/cm with distance increasing from 5 μm to 30 μm and was independent of the distance for larger *L* values. The inset in Figure 7 shows the optical microscope images of a Schottky diode before and after breakdown. One can see that the inner contact is mostly damaged. Lateral breakdown occurs close to the inner Schottky contact, where the electric field has its maximum. A similar reverse breakdown field dependence on the distance between two ohmic contacts fabricated on the T-HEMT with locally removed 2DEG was reported previously in Reference [18]. There, the critical breakdown field values reached 2 MV/cm for a short distance of *L* = 5 µm between two isolated devices. In our work, realistic devices—SBDs—were investigated in the reverse bias regime, demonstrating similar behaviour for the breakdown field with maximum values close to 0.8 MV/cm for the short distance (5 µm) between Schottky and ohmic contacts. Therefore, we conclude that the actual breakdown field is higher than 0.8 MV/cm and the absence of the thick GaN buffer does not deteriorate the breakdown characteristics by much.

### 3.2. Performance of T-HEMTs

**Figure 8:**
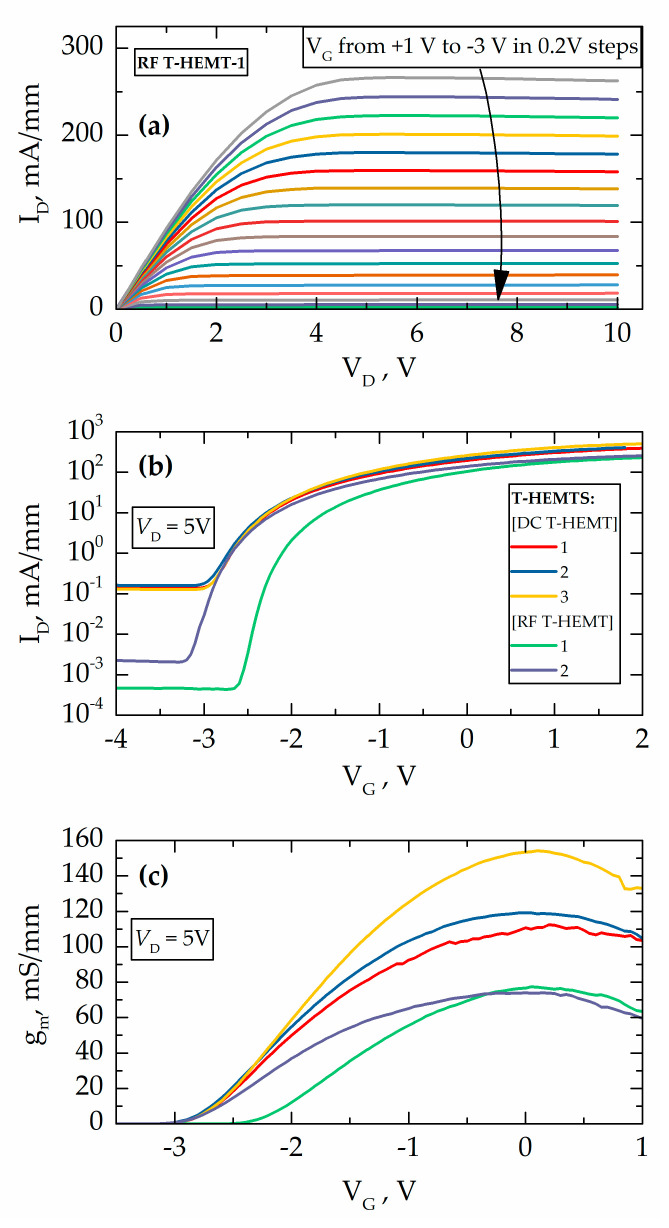
DC characteristics of T-HEMTs under study: (**a**) DC output characteristics of 0.4 mm wide RF T-HEMT-1 and comparisons of transfer (**b**) and transconductance (**c**) characteristics of the RF T-HEMTs and DC T-HEMTs with various values of the channel widths *W_Ch_*. The gate length for all devices is 5 µm.

Typical DC characteristics of representative T-HEMT are shown in Figure 8. As seen in Figure 8a, RF T-HEMT demonstrated drain current saturation at the level of 266 mA/mm under DC biases of *V_D_* = 10 V and *V_G_* = +1 V. This translates into an input power value of 2.6 W/mm for T-HEMT with a channel width of 0.4 mm. The drain current in the saturation region fell by 1–2% only. This indicates the advantages of efficient heat removal from the 2DEG channel in AlGaN/GaN with AlN NL that exploits the absence of the buffer layer and high thermal conductivity of the SiC substrate.

The transfer and transconductance (*g_m_*) characteristics at *V_D_* = 5 V for various T-HEMTs are shown in Figure 8b,c. The impact of mesa on the device performance can be identified from the transfer characteristics. Indeed, the circular DC T-HEMT devices demonstrated up to two orders of magnitude larger leakage currents in comparison to those measured for RF T-HEMTs. Both the maximum drain current and the transconductance values were found to be higher for the DC T-HEMTs demonstrating values up to 507 mA/mm and 154 mS/mm, respectively. Meanwhile, RF T-HEMTs demonstrated only 266 mA/mm and 77 mS/mm. The pinch-off region is observed beyond a gate bias of −3 V, which is in good agreement with *V_po_* obtained from *C*-*V* measurements.

One of the most effective ways to evaluate the quality of the material and the deep level traps is the low-frequency noise measurements. It is well known that low-frequency noise may differ significantly for the devices with almost identical DC characteristics. Elevated noise level is an indication of lower quality of the material, higher concentration of the deep level traps, lower reliability, and reduced lifetime of the devices. In the majority of cases, the low-frequency noise in field effect transistors complies with the McWhorter model [32,33]. In accordance with the model, the 1/*f* low-frequency noise is a result of tunnelling of the carriers to the layers adjacent to the channel. The model allows for estimation of the effective trap density responsible for noise, which is a good figure of merit for the noise level and overall quality of the material.

**Figure 9:**
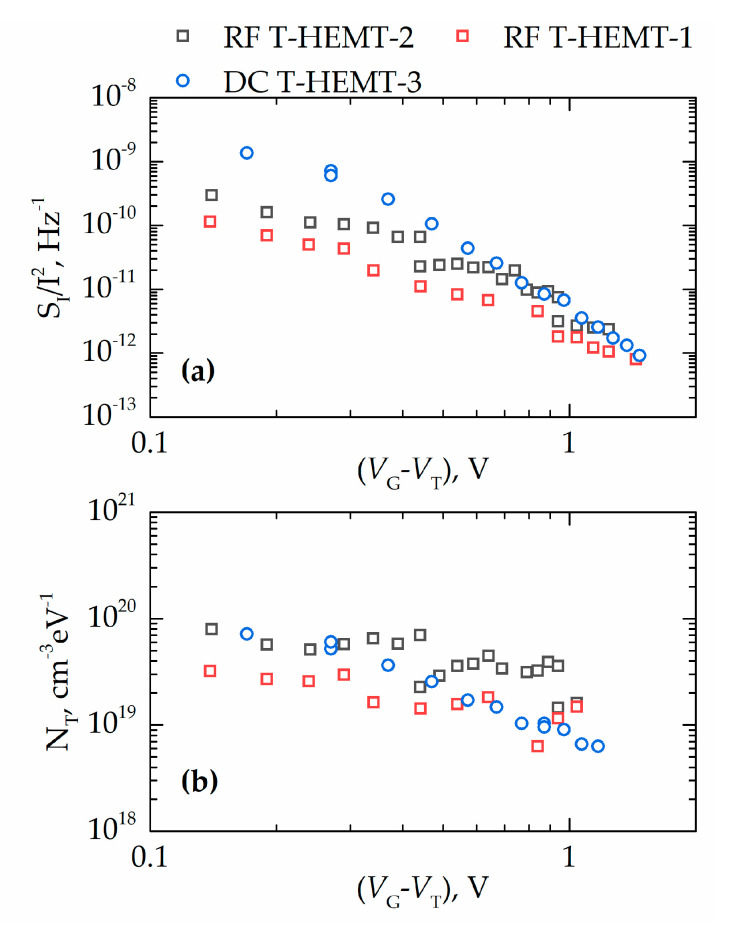
(**a**) Drain current noise *S_I_*/*I*^2^ at frequency *f* = 10 Hz for T-HEMTs of different channel widths ranging from 0.2 mm to 0.6 mm and (**b**) the effective trap density *N_T_* as a function of the gate voltage swing (*V_G_-V_T_*) for the same transistors.

The spectra of the drain current fluctuations had the form of 1/*f^γ^* noise with exponent *γ* = 0.9–1.1. The dependences of the noise *S_I_*/*I*^2^ on the gate voltage swing (*V_G_-V_T_*) at *f* = 10 Hz for three representative devices are shown in Figure 9a (here, *V_T_* is the threshold voltage determined from the transfer current voltage characteristics in the linear regime). As seen, noise depends on the gate voltage as (*V_G_-V_T_*)^2^ or steeper. It is known that, in many cases, this dependence at high gate voltages may become flat, indicating a contribution of the contact noise. It is seen from Figure 9a that this is not the case for the studied devices and that contacts do not contribute to noise significantly. The effective trap density *N_T_* in the McWhorter model can be estimated from gate voltage noise as follows [9]:

(4) $$S_{V_{G}}=\frac{S_{I}/I^{2}}{{\left(g_{m}/I\right)}^{2}}$$

(5) $$S_{V_{G}}=\frac{kTN_{T}e^{2}}{\gamma fW_{Ch}L_{G}C^{2}},$$

where *k* is the Boltzmann constant, *T* is the temperature, *W_Ch_* and *L_G_* is the channel area, *C* is the gate capacitance per unit area, and *γ* is the attenuation coefficient of the electron wave function under the barrier, taken to be 10^8^ cm^−1^.

According to the McWhorter model, input gate voltage noise does not depend on access resistance and carrier concentration in the channel [9]. The dependence of the effective trap density on the gate voltage in Figure 9b can be attributed to the dependence of the trap density on energy. The number of traps in this T-HEMT structure was found to be in the range 10^19^–10^20^ cm^−3^ eV^−1^. Some of the devices demonstrated *N_T_* < 10^19^ cm^−3^ eV^−1^. These values are of the same order or even smaller than those reported earlier for AlGaN/GaN HEMTs with a thick buffer layer [9]. Therefore, we conclude that studied T-HEMTs are characterized by the same quality as or even better quality than regular devices with thick buffers.

**Figure 10:**
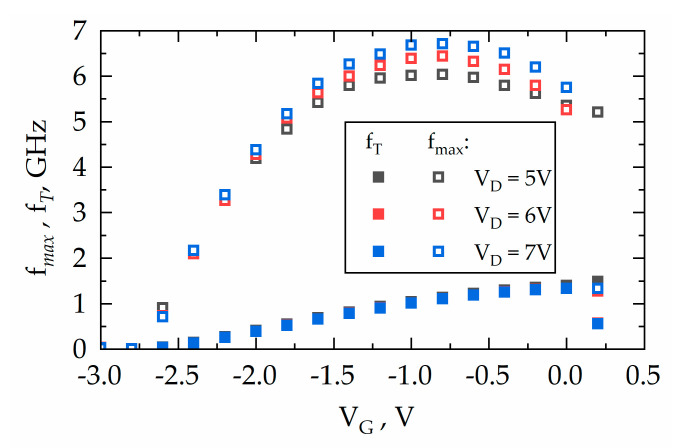
Frequencies *f_T_* and *f_max_* at different biasing conditions extracted from S-parameters measurements of RF T-HEMT-1 with *W_Ch_* = 0.4 mm and *L_G_* = 5 μm.

The unity current gain cut-off frequency (*f_T_*) and the unity maximum unilateral power gain frequency (*f_max_*) were found at various voltages down to the threshold voltage. The results are shown in Figure 10. The RF T-HEMTs with a 0.4-mm channel width demonstrated the highest operational frequencies, with values reaching *f_T_* = 1.33 GHz at *V_GS_* = 0 V with *V_D_* = 5 V and *f_max_* = 6.7 GHz at the bias of *V_G_* = −0.8 V and *V_D_* = 7 V. These results revealed a figure of merit (FOM) factor *f_T_* × *L_G_* up to 6.7 GHz × µm, which is comparable with the best value of 9.2 GHz × µm reported for the T-HEMTs in Reference [19]. The performance of RF T-HEMTs can be further improved in our processing via optimization of ohmic contact/access resistance and the reduction of channel length *L_SD_* in tandem with gate length *L_G_* [34,35]. Note that there is up to 3 times difference between the FOM factor of T-HEMTs and that of standard HEMTs, which requires more detailed investigations in the future [36].

## 4. Conclusions

AlGaN/GaN SBDs and HEMTs without GaN buffer layers have been fabricated on SiC substrates. 2DEG densities of 1 × 10^13^ cm^2^ with mobility of 1.7 × 10^3^ cm^2^/V∙s and 1.0 × 10^4^ cm^2^/V∙s at 300 K and 77 K, respectively, were found from the Hall measurements. The unterminated and unpassivated SBDs fabricated on these heterostructures exhibited high breakdown voltages up to −780 V, with the critical breakdown field reaching 0.8 MV/cm. Transistors on these heterostructures, so-called T-HEMTs, demonstrated maximum current density and transconductance values up to 0.5 A/mm and 150 mS/mm, respectively, with a negligible reduction in the drain current. This indicates improved thermal management due to a heterostructure design on the SiC substrate without a GaN buffer layer. By systematic low-frequency noise measurements, we estimated the effective trap density, which in T-HEMT structures was below the level of 10^19^ cm^−3^ eV^−1^. This value is similar to or even smaller than previously reported trap densities in heterostructures with thick GaN:C buffers. This means that avoiding a GaN:C buffer in GaN–AlN-SiC material does not lead to an increase in active (dislocation-related) trap density. The unity current gain cut-off and unity maximum unilateral power gain were measured to be 1.3 GHz and 6.7 GHz, respectively. Using this data, the figure of merit *f_T_* × *L_G_* is estimated at 6.7 GHz × µm. Therefore, we conclude that a buffer-free design did not compromise the quality of the structures or the performance of the devices. Our results confirm the potential of a GaN–SiC hybrid material for the development of HEMTs and SBDs for high-frequency and high-power applications with improved thermal stability.

## Figures and Tables

**Table 1:** Parameters of 2DEG in T-HEMT heterostructures at 300 K and 77 K.

	Hall Measurements		Simulation	Eddy Current Measurements
Parameter	300 K	77 K	300 K	300 K
*N*_2*DEG*_, ×10^13^ cm^−2^	1.00	0.96	1.0	-
*μ*_2*DEG*_, cm^2^/V∙s	1.7 × 10^3^	1.0 × 10^4^	-	-
*R_Sh_*, Ω/□	375	64	-	380 ± 10
